# Myalgia caused by chronic myositis associated with plasmacytosis: a case report

**DOI:** 10.1186/s12883-018-1123-3

**Published:** 2018-08-14

**Authors:** Taku Hatano, Masashi Takanashi, Hitoshi Tsuchihashi, Shin-Ichi Ueno, Arisa Hayashida, Yutaka Tsukune, Kazuaki Kanai, Yasushi Shimo, Nobutaka Hattori

**Affiliations:** 1 Department of Neurology, Juntendo University Graduate School of Medicine, 2-1-1 Hongo, Bunkyo-ku, Tokyo, 113-8421 Japan; 2 Department of Neurology, Juntendo Koshigaya Hospital, 560 Fukuroyama, Koshigayashi, Saitama 343-0032 Japan; 3 Department of Dermatology, Juntendo University Graduate School of Medicine, 2-1-1 Hongo, Bunkyo-ku, Tokyo, 113-8421 Japan; 4 Department of Internal Medicine, Division of Hematology, Juntendo University Graduate School of Medicine, 2-1-1 Hongo, Bunkyo-ku, Tokyo, 113-8421 Japan; 5 Department of Research and Therapeutics for Movement Disorders, Juntendo University Graduate School of Medicine, 2-1-1 Hongo, Bunkyo-ku, Tokyo, 113-8421 Japan

**Keywords:** Plasmacytosis, Chronic myositis, Myalgia, Autoimmune disease, Lymphoproliferative disease

## Abstract

**Background:**

Cutaneous and systemic plasmacytosis are skin disorders characterized by cutaneous polyclonal plasma cell infiltration accompanied by polyclonal hypergammaglobulinemia. Cutaneous plasmacytosis involvement is limited to the skin, mainly on the face and trunk, while systemic plasmacytosis also involves 2 or more organ systems. However, there have been no reports of inflammatory myositis due to plasmacytosis. Here, we report a patient with plasmacytosis who developed myalgia and easy fatigability due to inflammatory myositis.

**Case presentation:**

A 54-year-old man with cutaneous plasmacytosis on the face, chest, and back complained of a history of atypical facial and lower leg pain and easy fatigability since the age of 45 years. Muscle-strength tests revealed bilateral trivial gastrocnemius weakness with myalgia. The results of routine blood analysis, including creatine kinase and thyroid function, were normal, but levels of several inflammation markers and autoantibodies were elevated. Additionally, lower leg magnetic resonance imaging and gastrocnemius muscle biopsy revealed inflammatory myositis mimicking polymyositis. His plasmacytosis, myalgia, and lower leg weakness were ameliorated by prednisolone.

**Conclusion:**

The patient was diagnosed with inflammatory myositis due to plasmacytosis. Given that plasmacytosis has previously been reported to disrupt the immune status, myositis in this patient might have been associated with abnormal autoimmune inflammation. Neurologists and physicians should thus be aware that plasmacytosis might be associated with inflammatory myositis accompanied by myalgia.

## Background

Cutaneous and systemic plasmacytosis are skin disorders characterized by cutaneous polyclonal plasma cell infiltration accompanied by polyclonal hypergammaglobulinemia [1]. Although plasmacytosis is a rare condition, its prevalence in the Japanese population is relatively high [2–7]. Shimizu et al. reviewed 26 previously published cases with this disease, and found that all but two patients were Japanese [2]. But the precise prevalence of the disease in Japan, and other countries, remains unclear. Plasmacytosis has 2 different clinical phenotypes: cutaneous plasmacytosis, which is limited to the skin and characterized by reddish-brown plaques, mainly on the face and trunk; and systemic plasmacytosis, which involves 2 or more organ systems [1]. However, the association between systemic plasmacytosis and muscle involvement has not been reported. Here, we describe a patient with cutaneous plasmacytosis who complained of myalgia followed by the emergence of blown plaques on his face and trunk. Although serum studies failed to detect any elevation of creatinine kinase, magnetic resonance imaging (MRI) revealed abnormalities of the lower leg muscles. Histological analysis of a leg muscle biopsy revealed inflammatory myositis.

## Case presentation

A 54-year-old man with cutaneous plasmacytosis of the face, chest, and back consulted the neurology service at our hospital with complaints of tingling pain in his face, tenderness in the bilateral lower legs, and easy fatigability. He had developed red-brown plaques on his face and trunk at age 45 years, and consulted his local hospital at age 47. Skin biopsy was performed at that time and histological examination revealed characteristic findings of cutaneous plasmacytosis. At about the same time as he was diagnosed with skin plasmacytosis, the patient also complained of myalgia with easy fatigability and was diagnosed with fibromyalgia. His other medical history was remarkable for hypertension, angina pectoris, left mastoiditis, lumbar stenosis, and osteoarthritis of bilateral knees. Nine years after the onset of myalgia, the patient consulted our neurological service and was admitted to our department to identify the cause of facial and lower leg pain.

**Fig. 1:**
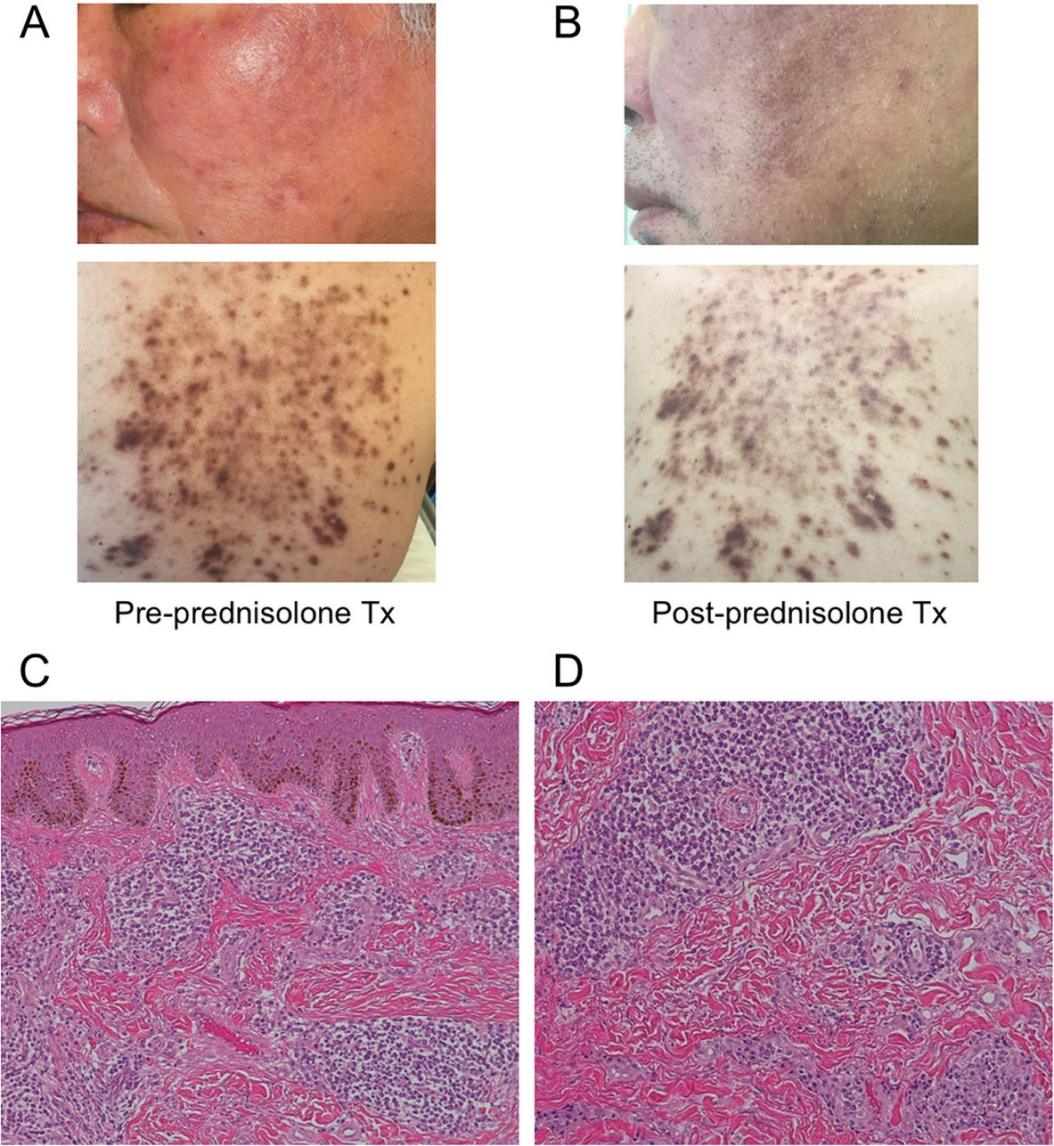
Cutaneous plasmacytosis in the patient. The patient presented with red-brown plaques on his face and trunk (**a**) which were ameliorated by prednisolone therapy. Although the pigmentation of facial lesions remained, redness and swelling improved to some extent (**b**). Histological examination revealed characteristic findings of cutaneous plasmacytosis (**c**, **d**). There was no abnormality in either the stratum corneum or epidermis, but plasma cells infiltrated the dermis. Tx, treatment

**Fig. 2:**
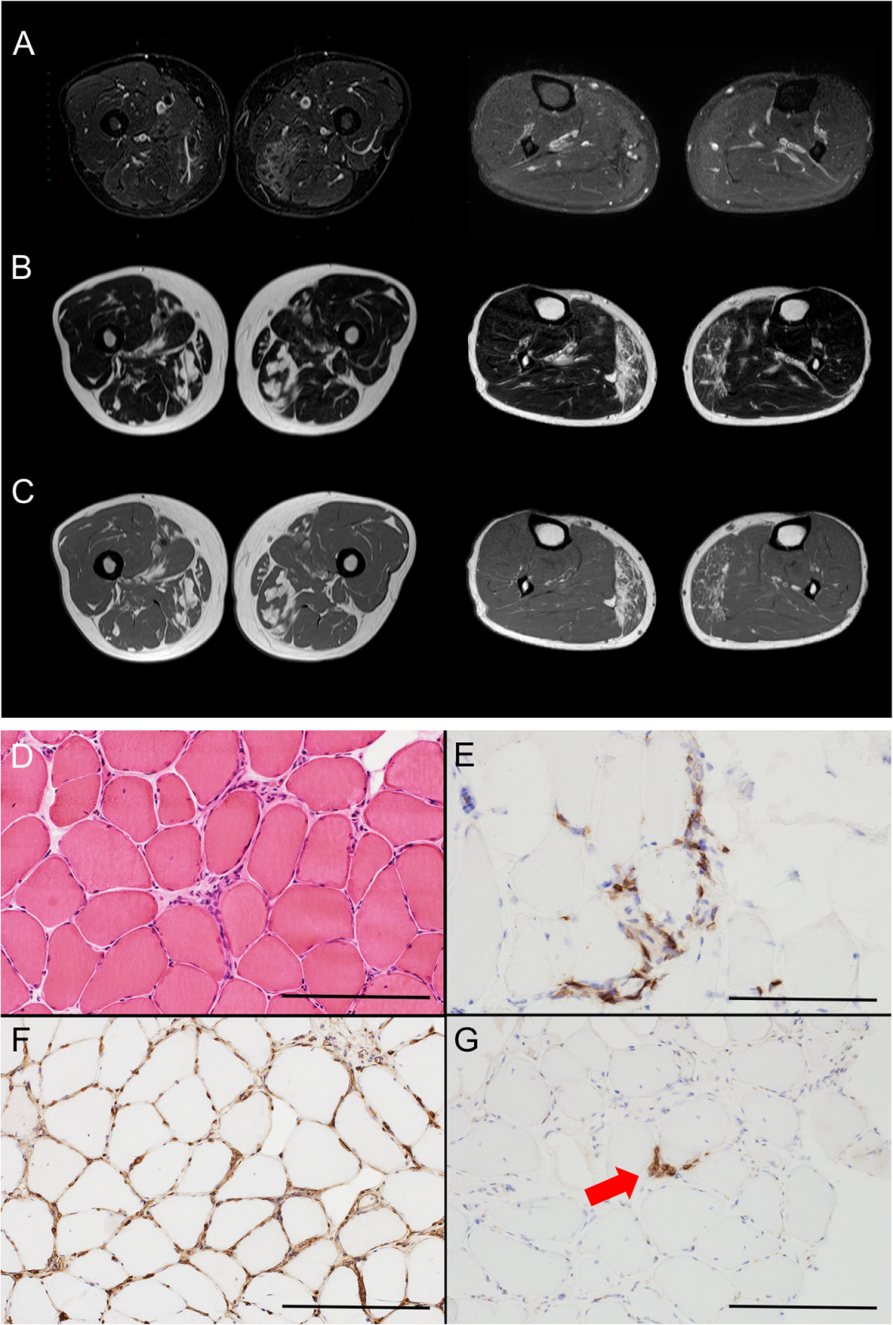
MRI and histological findings of myositis in the patient. Short tau inversion recovery (**a**), T2-weighted (**b**), and T1-weighted (**c**) MRI revealed inflammation of several muscles in the bilateral lower legs. **d**–**g** Gastrocnemius muscle pathology. Inflammatory cells invaded the endomysium (**d**, hematoxylin and eosin, bar 200 μm). Lymphocytes surrounding non-necrotic fibers were CD8+ cytotoxic T cells (**e**, CD8 immunostaining, bar 100 μm). Non-necrotic fibers showed ubiquitous MHC-class 1 antigen expression (F, MHC-class 1 immunostaining, bar 500 μm). A few plasmacytes were seen in the endomysium (**g**, red arrow, CD138 immunostaining, bar 200 μm)

On admission, the patient had red-brown plaques on his face and trunk (Fig. 1a). He complained of myalgia in his bilateral vastus lateralis muscles, hip adductors, and gastrocnemius, which worsened with pressure. Muscle strength tests revealed bilateral trivial weakness of the gastrocnemius with normal muscular tone. The results of general and neurological examinations and routine blood analysis, including creatine kinase 95 U/L (normal range 57–240) and thyroid function, were all normal. Serum antibodies specific to syphilis, neurotrophic viruses, human immunodeficiency virus, and thyroid and autoimmune diseases, including interleukin-6 (IL-6), IgG4, antinuclear, anti-Sm, anti-mitochondria M2, anti-SS-A/SS-B, anti-Jo-1, anti-Scl-70, anti-acetylcholine receptor, myeloperoxidase-, and proteinase 3-anti-neutrophil cytoplasmic antibodies were all normal. Rheumatoid factor and anti-cardiolipin antibodies were positive, but anti-citrullinated protein antibody and lupus anticoagulant were negative. Serum levels of soluble IL-2 receptor 873 U/mL (normal range 145–519), IgG 1914 mg/dL (normal range 870–1700) and IgA 619 mg/dL (normal range 110–410) were all elevated. However, both Bence Jones protein and M protein were negative. We performed a repeat skin biopsy to confirm the diagnosis of plasmacytosis. The biopsy specimen (Fig. 1c, d) showed prominent dermal perivascular proliferation of mature plasma cells, with a normal gamma chain kappa/lambda ratio, and a MIB-1-labeling index of less than 5%. In the specimen, CD79a expression was positive but CD56, cyclin D1, and Epstein Barr virus-encoded RNA were negative. There was no evidence of immunoglobulin heavy-chain gene rearrangements. Therefore, these findings were compatible with cutaneous plasmacytosis, not multiple myeloma. The findings of a nerve conduction study were all normal, but gastrocnemius needle electromyography revealed positive sharp waves, repetitive discharge, and polyphasic motor unit potentials. However, there was no evidence of early recruitment. These findings suggested denervation, but myogenic changes were inconclusive. T1- and T2-weighted MRI of the lower legs revealed abnormal high intensity in several muscles, including bilateral adductors, quadriceps, semimembranosus, and gastrocnemius muscles. Short tau inversion recovery imaging also showed abnormal intensity of the bilateral quadriceps and gastrocnemius muscles (Fig. 2a–c). These findings indicated myositis with chronic inflammation, and we therefore performed a biopsy of the right gastrocnemius muscle. Morphological analysis of the muscle specimen showed fiber size variability, scattered necrotic and regenerated fibers, and lymphocyte infiltration predominantly in the endomysium rather than the perimysium or the vessels (Fig. 2d), but no perifascicular atrophy or rimmed vacuoles. CD8+ toxic T cells surrounded the non-necrotic fibers and major histocompatibility complex (MHC) class 1 antigen expression was ubiquitous (Fig. 2e, f). There were almost no B cells, CD4+ cells, macrophages, or membrane attack complexes around the small vessels. A few plasmacytes were visible in the endomysium (Fig. 2g), and they stained negative for IgG4. These pathological features were compatible with European Neuromuscular Center classification criteria of polymyositis [8].

The patient’s muscle pain was unaffected by acetaminophen and pregabalin, but his myalgia and cutaneous plasmacytosis on his face and back were ameliorated by prednisolone (20 mg/day) (Fig. 1b).

## Discussion and conclusion

Plasmacytosis can be either localized, as in plasma-cell type giant lymph node hyperplasia, or systemic [1]. The current patient showed multiple reddish-brown skin lesions, typical of cutaneous plasmacytosis, scattered over his face and back. Serum and dermal pathological analyses revealed elevated levels of IgG and IgA, without Bence Jones or M protein, and proliferation of mature plasma cells in the dermis. These findings suggested plasmacyotosis rather than multiple myeloma. The abnormally high intensity of muscles in T1- and T2-weighted MRI indicated chronic myositis with fatty degeneration. The needle electromyography failed to provide conclusive evidence for myogenic changes, although the results might be influenced by the neurogenic changes from lumbar stenosis. Plasmacytosis occasionally proliferates systemically, similar to the skin. However, although several previous reports have described the involvement of superficial lymph nodes, liver, spleen and lung [1, 5, 6], there have been no reports of an association between proliferating plasma cells and myopathy. Muscle biopsy in the current case showed proliferation of lymphocytes, but not plasma cells, suggesting that the myositis in this case might have been related to chronic inflammation, rather than to the invasion of plasma cells into the muscles.

The proliferation of lymphocytes in the muscle suggested that the inflammation might be related to an autoimmune response. Pathological investigation indicated that the myositis could be a compatible finding of polymyositis. Inflammatory myopathies, such as polymyositis, dermatomyositis, and inclusion body myositis, are known to be associated with autoimmune disorders. Plasma cells are antibody-producing, terminally differentiated quiescent B cells [9], and abnormal plasma cells might thus perturb the immune system. Indeed, autoimmune disorders and lymphoproliferative diseases, including Castleman’s disease, chronic lymphocytic leukemia, and lymphoma, share a bidirectional relationship [10]. Dermatomyositis and polymyositis are occasionally associated with lymphoma development [10]. Additionally, Kiniwa et al. reported a patient with autoimmune hemolytic anemia due to cutaneous plasmacytosis [7]. Notably, serum examinations in the present case failed to reveal any abnormalities of myositis-associated biomarkers, including IL-6, anti-nuclear antibodies, or anti-Jo1 antibodies; however, he had abnormally high serum levels of soluble IL-2 receptor, rheumatoid factor, and anti-cardiolipin antibody, suggesting an immunological perturbation. In this context, we concluded that his myositis might have been caused by an immunological abnormality related to plasmacytosis. This was supported by the fact that his skin lesions and myalgia were ameliorated by prednisolone, but not by non-steroidal anti-inflammatory drugs. Skin lesions in most patients with plasmacytosis are resistant to several therapies, but the current case suggests that immunomodulation using steroids and cyclophosphamide might be an effective option [3], and that inflammation might play an important role in the pathogenesis of skin lesions in patients with plasmacytosis.

We should consider some of the shortcomings of our case assessment. The patient’s myositis could be considered an idiopathic inflammatory myositis, including polymyositis, dermatomyositis, and inclusion body myositis, incidentally combined with plasmacytosis. Prednisolone and immunomodulation therapy also have an effect on idiopathic inflammatory myositis. Additionally, the patient was not examined for the presence of several autoimmune inflammatory myositis-associated antibodies, except for anti-Jo1 antibody. However, his myositis showed atypical findings of idiopathic inflammatory myositis, such as very slow progression without rimmed vacuole, no elevation of creatine kinase, and no characteristic skin lesions in dermatomyositis. Therefore, we believe that the myositis was associated with plasmacyotosis.

To the best of our knowledge, this is the first report of a patient with inflammatory myositis mimicking polymyositis and plasmacytosis. Physicians and neurologists should be aware of the co-existence of plasmacytosis and inflammatory myositis.

## Abbreviations

Ig: Immunoglobulin
IL-2: Interleukin-2
IL-6: Interleukin-6
MHC: Major histocompatibility complex
MRI: Magnetic resonance imaging; Tx, treatment
